# Activation of PKG and Akt Is Required for Cardioprotection by Ramelteon-Induced Preconditioning and Is Located Upstream of mKCa-Channels

**DOI:** 10.3390/ijms21072585

**Published:** 2020-04-08

**Authors:** Carolin Torregroza, Osameh Jalajel, Annika Raupach, Katharina Feige, Sebastian Bunte, André Heinen, Alexander Mathes, Markus W. Hollmann, Ragnar Huhn, Martin Stroethoff

**Affiliations:** 1 Department of Anesthesiology, University Hospital Duesseldorf, Moorenstr. 5, 40225 Duesseldorf, Germany; Carolin.Torregroza@med.uni-duesseldorf.de (C.T.); Osameh.Jalajel@hhu.de (O.J.); Annika.Raupach@med.uni-duesseldorf.de (A.R.); KatharinaKristina.Feige@med.uni-duesseldorf.de (K.F.); buntesebastian@gmail.com (S.B.); Martin.Stroethoff@med.uni-duesseldorf.de (M.S.); 2 Department of Anesthesiology, Amsterdam University Medical Center (AUMC), Location AMC, Meiberdreef 9, 1105 AZ Amsterdam, The Netherlands; M.W.Hollmann@amsterdamumc.nl; 3 Department of Internal Medicine, Elbe Clinics Stade-Buxtehude, Bremervoerder Str. 111, 21682 Stade, Germany; 4 Institute of Cardiovascular Physiology, Heinrich-Heine-University Duesseldorf, Universitaetsstr. 1, 40225 Duesseldorf, Germany; Andre.Heinen@hhu.de; 5 Department of Anesthesiology and Intensive Care Medicine, University Hospital Cologne, Kerpener Str. 62, 50937 Cologne, Germany; Alexander.Mathes@uk-koeln.de

**Keywords:** Ramelteon, myocardial infarction, preconditioning, PKG, Akt

## Abstract

Ramelteon is a Melatonin 1 (MT1)—and Melatonin 2 (MT2)—receptor agonist conferring cardioprotection by pharmacologic preconditioning. While activation of mitochondrial calcium-sensitive potassium (mK_Ca_)-channels is involved in this protective mechanism, the specific upstream signaling pathway of Ramelteon-induced cardioprotection is unknown. In the present study, we (1) investigated whether Ramelteon-induced cardioprotection involves activation of protein kinase G (PKG) and/or protein kinase B (Akt) and (2) determined the precise sequence of PKG and Akt in the signal transduction pathway of Ramelteon-induced preconditioning. Hearts of male Wistar rats were randomized and placed on a Langendorff system, perfused with Krebs–Henseleit buffer at a constant pressure of 80 mmHg. All hearts were subjected to 33 min of global ischemia and 60 min of reperfusion. Before ischemia, hearts were perfused with Ramelteon (Ram) with or without the PKG or Akt inhibitor KT5823 and MK2206, respectively (KT5823 + Ram, KT5823, MK2206 + Ram, MK2206). To determine the precise signaling sequence, subsequent experiments were conducted with the guanylate cyclase activator BAY60-2770 and the mK_Ca_-channel activator NS1619. Infarct size was determined by 2,3,5-triphenyltetrazolium chloride (TTC) staining. Ramelteon-induced infarct size reduction was completely blocked by KT5823 (*p* = 0.0012) and MK2206 (*p* = 0.0005). MK2206 with Ramelteon combined with BAY60-2770 reduced infarct size significantly (*p* = 0.0014) indicating that PKG activation takes place after Akt. Ramelteon and KT5823 (*p* = 0.0063) or MK2206 (*p* = 0.006) respectively combined with NS1619 also significantly reduced infarct size, indicating that PKG and Akt are located upstream of mK_Ca_-channels. This study shows for the first time that Ramelteon-induced preconditioning (1) involves activation of PKG and Akt; (2) PKG is located downstream of Akt and (3) both enzymes are located upstream of mK_Ca_-channels in the signal transduction pathway.

## 1. Introduction

Cardioprotective interventions such as preconditioning—ischemic or pharmacological—are powerful measures to protect the heart against myocardial ischemia–reperfusion injury [1,2]. Preconditioning can be induced by different stimuli, such as short cycles of ischemia followed by reperfusion (ischemic preconditioning) [1], whereas pharmacological preconditioning is realized with various drugs such as volatile anesthetics [3], opioids [4], or noble gases [5].This pharmacological preconditioning effect can also be induced by activation of Melatonin receptors (MT) [6].

Ramelteon is a Melatonin receptor agonist with high affinity to MT1- and MT2-receptors but less affinity to MT3-receptors [7]. Previously, we demonstrated that Ramelteon confers cardioprotection by preconditioning and that activation of mitochondrial calcium-sensitive potassium (mK_Ca_)-channels is involved [8]. Furthermore, activation of these channels by Ramelteon led to a release of radical oxygen species with downstream inhibition of the mitochondrial permeability transition pore (mPTP) [9]. However, the signaling pathway of Ramelteon-induced preconditioning upstream of mitochondrial potassium channels and downstream of Melatonin receptors is unknown. It has been shown that the guanylate cyclase (GC)-cyclic guanosine monophosphate (cGMP)-protein kinase G (PKG) pathway seems to play a crucial role in Melatonin-induced cardioprotection [10] and PKG is an upstream regulator of mK_Ca_-channels [11,12,13]. Activation of the serine-threonine kinase Akt was shown to be cardioprotective against ischemia–reperfusion injury [14,15] and was also shown in the context of pharmacological preconditioning, e.g., with the opioid receptor agonist remifentanil [16]. Das et al. showed that sildenafil, a phosphodiesterase-5 (PDE-5) inhibitor, also led to phosphorylation of Akt [17]. Inhibition of PDE-5 increases the level of cGMP and activates cGMP-dependent protein kinase G. Thus, both enzymes are influenced by sildenafil, a drug for which cardioprotective properties by preconditioning have also been demonstrated [13]. 

So far, the Ramelteon-induced protective signaling pathway upstream of mK_Ca_-channels is completely unknown. Therefore, the aim of this study was to investigate whether and in which sequence PKG and/or Akt are involved in the signaling pathway of Ramelteon-induced cardioprotection and if they are located upstream of mK_Ca_-channels.

## 2. Results

### 2.1. Animal Characteristics

There were no differences in body weight, wet and dry heart weight, and level or time of maximal ischemic contracture in both parts of the study (Table 1).

### 2.2. Infarct Size—Part 1

**Figure 1:**
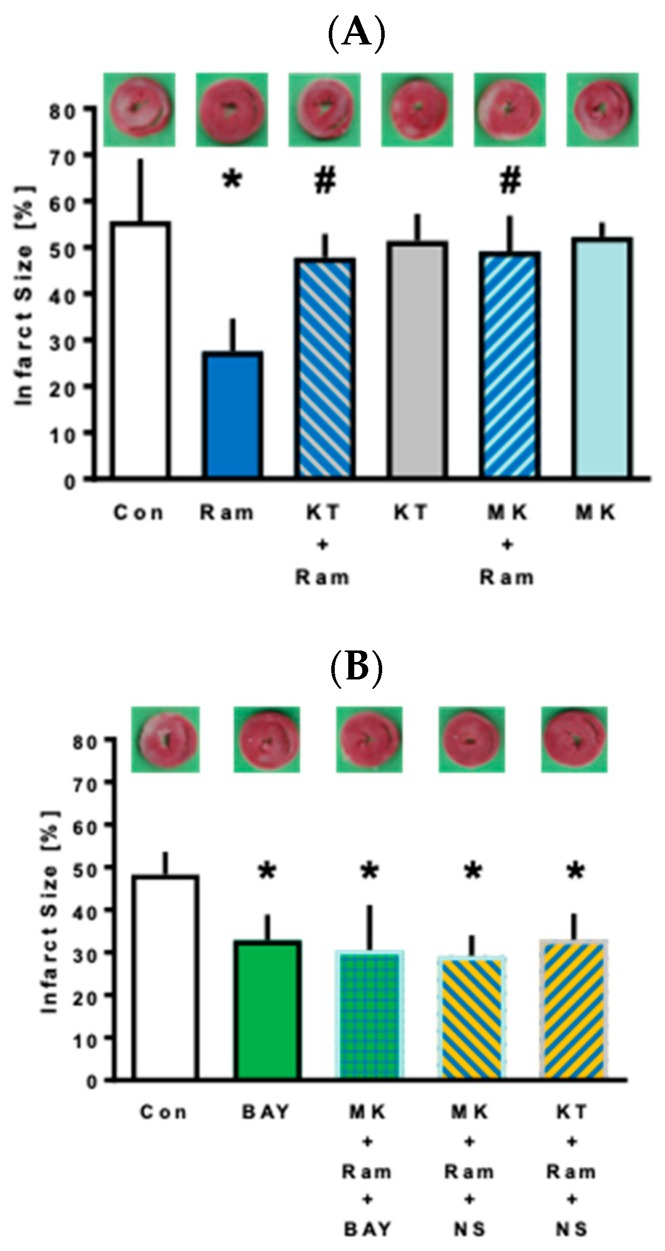
Infarct size measurement. Histogram shows the infarct size of part 1 (A) and part 2 (B) of the study. Data are presented as means ± SD, (**A**) * *p* < 0.0001 vs. Con, # *p* = 0.0012 KT + Ram vs. Ram, # *p* = 0.0005 MK + Ram vs. Ram. (**B**) * *p* = 0.0057 BAY vs. Con, * *p* = 0.0014 MK + Ram + BAY vs. Con, * *p* = 0.0006 MK + Ram + NS vs. Con, * *p* = 0.0063 KT + Ram + NS vs. Con.

Infarct size of the control group was 56% ± 14% and was reduced by Ramelteon to 28% ± 7% (*p* < 0.0001 vs. Con, Figure 1A). The respective inhibitor of PKG and Akt completely abolished cardioprotection by Ramelteon (KT + Ram: 48% ± 5%, *p* = 0.0012 vs. Ram and MK + Ram: 49% ± 8%, *p* = 0.0005 vs. Ram, Figure 1A). Both blockers did not have any effect on infarct size itself (KT: 51% ± 6%, *p* = 0.9311 vs. Con and MK: 52% ± 3%, *p* = 0.9734 vs. Con, Figure 1A).

### 2.3. Infarct Size—Part 2

The soluble guanylate cyclase (sGC) activator BAY60-2770 reduced infarct size from 48% ± 5% to 33% ± 6% (*p* = 0.0057 vs. Con, Figure 1B). Application of BAY60-2770 in combination with Ramelteon and the Akt inhibitor MK2206 showed an infarct size of 31% ± 11% (*p* = 0.0014 vs. Con), indicating that PKG might be located downstream of Akt in the signal transduction pathway of Ramelteon preconditioning. Combination of MK2206 and Ramelteon with the mK_Ca_-channel activator NS1619 reduced infarct size to 29% ± 5% (*p* = 0.0006 vs. Con). Furthermore, the combination of KT5823 and Ramelteon with NS1619 led to a significant infarct size reduction (KT+Ram+NS: 33% ± 6%; *p* = 0.0063 vs. Con), indicating that PKG and Akt, respectively, are located upstream of mK_Ca_-channels.

### 2.4. Cardiac Function

Hemodynamic variables of both parts of the study are presented in Table 2 and Table 3. There were no differences in heart rate between the study groups in part one and part two of the study. During reperfusion, left ventricular developed pressure and coronary flow were significantly lower in all groups compared to baseline in both parts of the study. 

## 3. Discussion

Investigating the role of Akt and PKG in Ramelteon-induced cardioprotection, we demonstrated their crucial role and the—so far unknown—signal transduction cascade of activation in relation to mK_Ca_-channels. Akt activation takes place before PKG, and both are located upstream of mK_Ca_-channels. 

The present study continues our research on the cardioprotective properties of Ramelteon and its underlying cardioprotective mechanism. In contrast to Melatonin, Ramelteon, which is clinically used for the treatment of insomnia, is a highly specific MT-receptor agonist without affecting other types of receptors [18]. Recently, we demonstrated that Ramelteon-induced preconditioning reduces infarct size in a concentration-dependent manner and involves activation of mK_Ca_-channels [8]. The activation of mK_Ca_-channels inhibits opening of the mitochondrial permeability transition pore (mPTP) as a key element in the underlying mechanism of myocardial protection [19,20]. There is evidence that mK_Ca_-channels are located downstream in the signal transduction cascade of preconditioning. Nevertheless, upstream signaling with involvement of PKG and Akt in Ramelteon-induced cardioprotection remains unknown. 

The data demonstrate that PKG and Akt are involved in the cardioprotective effect induced by Ramelteon as both the PKG inhibitor KT5823 and the Akt inhibitor MK2206 completely abolished the infarct size reduction. KT5823 is a potent, highly specific, cell-permeable inhibitor of PKG with an IC_50_ 60 nM and a K_i_ value of 234 nM [21,22]. Both PKA (K_i_ 4 µM) and PKC (K_i_ >10µM) are only influenced by KT5823 at significantly higher concentrations than those used in our experiments. MK2206 is a highly selective allosteric inhibitor of all three Akt isoforms (Akt1, Akt2, and Akt3), binding the interface of the catalytically active kinase domain and the regulatory domain, locking the kinase in an inactive conformation [23]. No inhibitory effect on other protein kinase can be found in the literature.

Furthermore, the data of the second part of our study show that Akt is activated before PKG, since the administration of the PKG activator BAY60-2770 and the simultaneous blockade of Akt led to a significant infarct size reduction (see Figure 1B, 3rd bar). In the case of an opposite order, this combination would result in a loss of Ramelteon-induced protection due to the direct effect of Akt inhibition on reduction of infarct size. Nitric-oxide-independent sGC agonists such as BAY60-2770 are valuable tools to examine the NO–cGMP-PKG signaling pathway. Several previous studies have used BAY60-2770 to stimulate sGC and have shown that PKG activity increases consecutively without increasing PKG expression [24,25,26]. Lee et al. also showed that tissue samples of BAY60-2770-perfused isolated Langendorff hearts had two-fold higher cGMP levels and increased PKG activity [27].

Our results clearly indicate that PKG and also Akt are located upstream of these mK_Ca_-channels, as application of the mK_Ca_-channel activator NS1619 in the presence of the PKG inhibitor KT5823 and the Akt inhibitor MK2206 respectively reduced infarct size significantly (see Figure 1B, 4th and 5th bar). Deenadayalu et al. showed a direct link between PKG and mK_Ca_-channels [11], e.g., by phosphorylation of PKG at serine 1072 of K_Ca_-channels [28]. It seems that the signaling pathway of Ramelteon-induced preconditioning shows distinct similarities to the signaling cascade of ischemic preconditioning. A described protective signaling pathway of ischemic preconditioning is the Reperfusion Injury Salvage Kinase (RISK) pathway, which mediates cardioprotection via, e.g., PI3K and Akt. Akt acts via endothelial nitric oxide synthase (eNOS) and nitric oxide (NO), and these molecules activate sGC and PKG [29]. Our results demonstrate that ischemia as a preconditioning stimulus as well as Ramelteon as a pharmacological preconditioning stimulus trigger similar cardioprotective signaling pathways. Furthermore, the mitochondria represent the end effector in the signaling cascade of cardioprotective interventions such as preconditioning [30]. In this context, mitochondrial potassium channels play a pivotal role, as activation of these channels is necessary to mediate the cardioprotective effect. In a previous study, we were able to show that Ramelteon-induced preconditioning is mediated via activation of mitochondrial potassium channels [8], again showing similarities to cardioprotection induced by ischemic preconditioning. Thus, Ramelteon seems to trigger cardioprotection in a similar manner known from ischemic preconditioning, and both types of interventions share common pathways. 

Although we see a strong effect on infarct size, no hemodynamic improvement during the reperfusion phase was detected between the groups. The exact reason for this is unclear, but the occurrence of myocardial stunning is often discussed, i.e., a temporary depression of function in the surviving myocardial tissue, especially after global ischemia. As global function of the left ventricle is measured, we cannot discriminate between effects that belong to differences in infarct size or differences in the degree of stunning. 

In our previous study, we demonstrated that activation of mK_Ca_-channels plays a pivotal role in Ramelteon-induced preconditioning [8]. Activation of mK_Ca_-channels leads to inhibition of mPTP opening. Cell death and loss of cardioprotection are the consequences of mPTP opening. Thus, inhibition of mPTP opening is a key step in the underlying signal cascade of myocardial protection [31]. 

**Figure 2:**
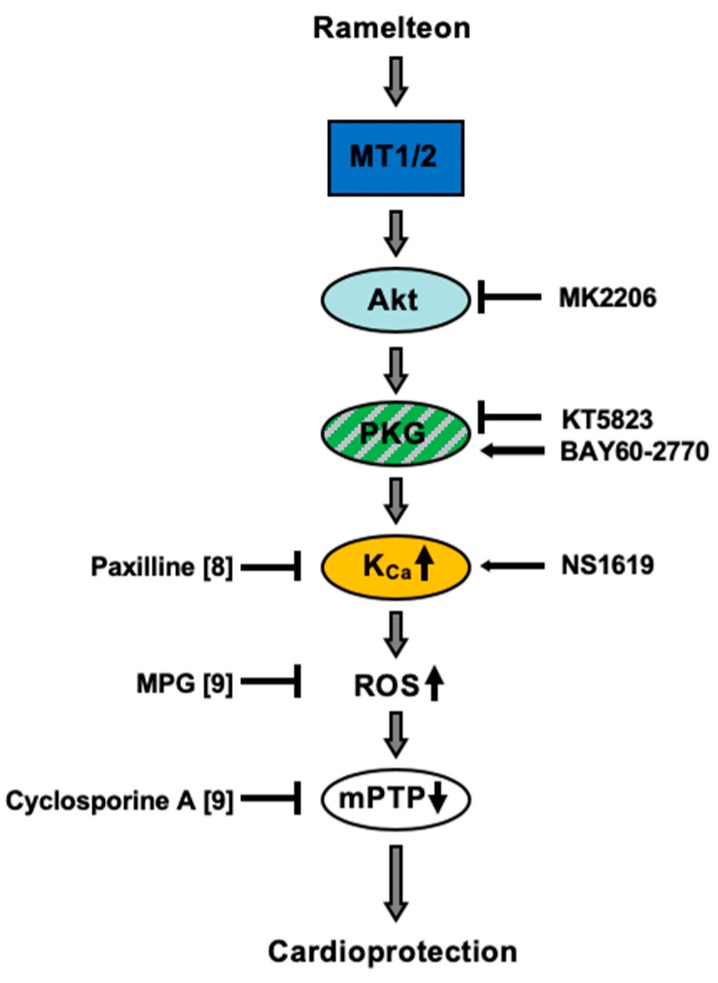
Possible signaling pathway of Ramelteon-induced cardioprotection against myocardial ischemia–reperfusion injury in rats. K_Ca_-channel = Ca^2+^-sensitive potassium (K_Ca_) channel; KT5823 = PKG inhibitor; BAY60-2770 = sGC activator; MK2206 = Akt inhibitor; NS1619 = K_Ca_-channel activator; Paxilline = K_Ca_-channel inhibitor; ROS = reactive oxygen species; MPG = ROS scavenger; mPTP = mitochondrial permeability transition pore; Cyclosporine A = mPTP inhibitor.

Following the signal transduction cascade downstream of mK_Ca_-channels, activation of these channels leads to inhibition of mPTP opening [32]. Inhibition of mPTP opening is triggered by reactive oxygen species (ROS) that are released by activation of mK_Ca_-channels [32]. Data from a previous study show that scavenging of ROS with the radical scavenger N-2-Mercaptopropionylglycine abrogated the infarct-size-reducing effect of Ramelteon, indicating an involvement of ROS in Ramelteon-induced cardioprotection [9]. These results show that inhibiting the mPTP with cyclosporine A, cardioprotection could be restored completely [9]. Taking together the results from our previous studies [8,9] and the data from the present study, we suggest the underlying signaling pathway for Ramelteon-induced preconditioning as shown in Figure 2. 

In addition to the role of mK_Ca_-channels in signal transduction of Ramelteon-induced preconditioning, they also take on an important function in relation to cardioprotection and comorbidities, e.g., diabetes and age. It was shown that diabetes [33] but also aging [34,35] abolished the infarct-size-reducing effect of preconditioning strategies. We previously demonstrated a protective effect of the aged rat heart by activating mK_Ca_-channels with NS1619 [34]. Thus, even in the aged heart where, e.g., ischemic preconditioning failed to induce infarct size reduction [36], cardioprotection can still be induced by activation of mK_Ca_-channels [34]. A possible effect of Ramelteon, for example, in connection with aging, must be investigated in further studies.

Furthermore, Melatonin receptors and their agonists seem to play a pivotal role in treatment of endothelial dysfunction and damage, which frequently occurs in elderly patients, leading to cardiovascular diseases. Focusing on the aspect of endothelial dysfunction, Nakao et al. investigated the effects of Melatonin on Angiotensin-II-induced vascular endothelial damage [37]. They suggested that both Melatonin and Ramelteon ameliorate Angiotensin-II-induced vascular endothelial damage via an antioxidative effect [37]. Taking together the involvement of mK_Ca_-channels in Ramelteon-induced preconditioning in relation to cardiovascular comorbidities as well as findings from Nakao et al. on vascular damage, Ramelteon seems to be a promising drug for potential clinical application. The relevance of Ramelteon-induced preconditioning in the context of comorbidities might be interesting to investigate in future studies. 

## 4. Materials and Methods 

This investigation was in accordance with the Guide for the Care and Use of Laboratory Animals published by the US National Institutes of Health (NIH Publication No. 85-23, revised 1996) and was approved from the Animal Ethics Committee of the University of Düsseldorf, Germany (O27/12). The experiments and reporting of the results were done in accordance with the ARRIVE guidelines.

### 4.1. Surgical Preparation

Surgical preparation was performed as described previously [13,34]. Briefly, male Wistar rats were anesthetized by intraperitoneal injection of 90 mg/kg pentobarbital and decapitated. After thoracotomy, the hearts were excised, mounted on a Langendorff system, and perfused at a constant pressure of 80 mmHg with a Krebs–Henseleit solution (pH 7.38–7.43), enriched with 95% O_2_ and 5% CO_2_. The solution contains (in mM): 118 NaCl, 4.7 KCl, 1.2 MgSO_4_, 1.17 KH_2_PO_4_, 24.9 NaHCO_3_, 2.52 CaCl_2_, 0.5 EDTA, 11 glucose, and 1 lactate at 37 °C. A fluid-filled balloon was inserted into the left ventricle, and the end-diastolic pressure was set at 2–8 mmHg. The hearts underwent an equilibration period of 20 min. We measured heart rate, left ventricular end-systolic pressure (LVESP), coronary flow, and left ventricular end-diastolic pressure (LVEDP) continuously and digitized it at a sampling rate of 500 Hz using an analogue to digital converter system (PowerLab/8SP, ADInstrument Pty Ltd., Castle Hill, Australia). Left ventricular developed pressure (LVDP) was calculated as LVESP–LVEDP. Data were continuously recorded on a personal computer using Chart for Windows v5.0 (ADInstruments Pty Ltd., Castle Hill, Australia). The maximal contracture as well as the time to maximal contracture was detected by analyzing the course of contracture development during ischemia and selecting the time point when contracture reached its highest level in each experiment. 

### 4.2. Experimental Protocol

**Figure 3:**
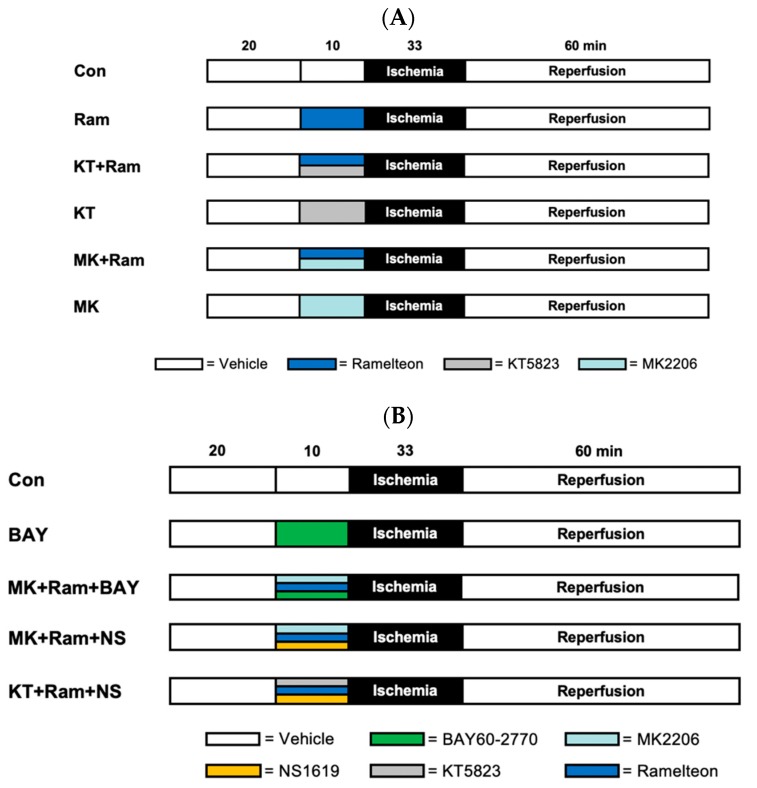
Experimental protocol Part 1 (**A**) and Part 2 (**B**). (**A**) Con = Control, Ram = Ramelteon, KT = KT5823 (PKG inhibitor), MK = MK2206 (Akt inhibitor). (**B**) Con = Control, BAY = BAY60-2770 (sGC Activator), Ram = Ramelteon, MK = MK2206 (Akt inhibitor), KT = KT5823 (PKG inhibitor).

The study was performed in two parts. The first part investigated whether PKG and/or Akt are involved in Ramelteon-induced preconditioning. Previously, we demonstrated that the lowest cardioprotective concentration of Ramelteon is 0.03 µM [8]. The concentration of the respective inhibitors of PKG (KT5823) and Akt (MK2206) was taken from the literature [13,23]. The inhibitors and Ramelteon were administered before global ischemia and reperfusion (Figure 3A). Each group underwent a baseline period of 20 min, 33 min of global ischemia, and 60 min of reperfusion, respectively. In all groups, global myocardial ischemia was induced by stopping the perfusion of the whole heart. Rat hearts were randomly assigned into six groups (*n* = 6 per group):

**Control (Con):** Hearts were perfused with Krebs–Henseleit solution for 10 min.
**Ramelteon (Ram):** Hearts were perfused with 0.03 µM Ramelteon for 10 min.
**KT5823+Ramelteon (KT + Ram):** Hearts were perfused with 1 µM KT5823 [13] combined with 0.03 µM Ramelteon for 10 min.
**KT5823 (KT):** To rule out an effect on myocardial infarction size by KT5823 itself, KT5823 was also administered for 10 min without Ramelteon.
**MK2206+Ramelteon (MK + Ram):** Hearts were perfused with 15 nM MK2206 [23] combined with 0.03 µM Ramelteon for 10 min.
**MK2206 (MK):** To rule out an effect on myocardial infarction size by MK2206 itself, MK2206 was also administered for 10 min without Ramelteon.

The second part of the study was designed to determine the precise sequence of PKG and Akt in the signal transduction pathway of Ramelteon-induced preconditioning (Figure 3B). The concentration of the activators (BAY60-2770 and NS1619) and inhibitors (MK2206 and KT5823) were taken from the literature [13,23,27]. The substances were administered before global ischemia and reperfusion rat hearts were randomly assigned to five experimental groups (*n* = 6 per group):

**Control (Con):** Hearts were perfused with Krebs–Henseleit solution for 10 min.
**BAY60-2770 (BAY):** Hearts were perfused with the soluble guanylate cyclase (sGC) activator BAY60-2770 (leading to activation of PKG) in a concentration of 5 nM for 10 min [27].
**MK2206+Ram+BAY60-2770 (MK+Ram+BAY):** Hearts were perfused with Ramelteon (0.03 µM) in combination with the Akt inhibitor MK2206 (15 nM) and the sGC activator BAY60-2770 (5 nM) for 10 min.
**MK2206+Ram+NS1619 (MK+Ram+NS):** Hearts were perfused with Ramelteon (0.03 µM) in combination with the Akt inhibitor MK2206 (15 nM) and the mK_Ca_-channel activator NS1619 (10 µM) for 10 min.
**KT5823+Ram+NS1619 (MK+Ram+NS):** Hearts were perfused with Ramelteon (0.03 µM) in combination with the PKG inhibitor KT5823 (1 µM) and the mK_Ca_-channel activator NS1619 (10 µM) for 10 min.

After reperfusion, hearts were cut into transverse slices, starting from the cardiac apex to just before the cardiac valvular plane. The slices were stained with 0.75% triphenyltetrazolium chloride (TTC) solution. The size of the infarcted area was determined by planimetry using SigmaScan Pro 5 computer software (SPSS Science Software, Chicago, IL, USA).

### 4.3. Statistical Analysis

#### 4.3.1. Sample Size Analysis

The calculated sample size was *n* = 6 for detecting a 25% mean difference in infarct size (power 80%, α < 0.05 (two-tailed)).

#### 4.3.2. Statistical Approach

All data are expressed as mean ± SD. Analysis of statistical data was performed using GraphPadStatMateTM (GraphPad Software, San Diego, CA, USA). Part one and part two of the study were analyzed separately. A researcher blinded to the experimental groups evaluated the infarct sizes. Infarct sizes were analyzed by one-way analysis of variance (ANOVA) followed by Tukey’s post hoc test. Comparisons of hemodynamic data among groups or among time points in a group were analyzed by two-way ANOVA followed by Tukey’s post hoc test for group effects and Dunnett´s post hoc test for time effects. Changes were considered statistically significant if *p* values were less than 0.05.

## 5. Conclusions

Our results demonstrate that the specific MT-receptor agonist Ramelteon involves activation of PKG and Akt for mediating its cardioprotective effect. Furthermore, we can show that activation of the respective elements of the underlying signaling cascade takes place in the following order: Akt–PKG–mK_Ca_-channel. Therefore, the current results are a further step toward clarifying the underlying mechanisms of Ramelteon-induced preconditioning.

## Figures and Tables

**Table 1:** Weights and ischemic contracture.

	*n*	Body Weight (g)	Heart Weight Dry (g)	Heart Weight Wet (g)	Time of Max. Ischemic Contracture (min)	Level of Max. Ischemic Contracture (mmHg)
Con	6	293 ± 15	0.14 ± 0.01	1.29 ± 0.06	16 ± 1	69 ± 11
Ram	6	289 ± 15	0.12 ± 0.02	1.30 ± 0.05	17 ± 2	64 ± 13
KT + Ram	6	282 ± 13	0.14 ± 0.01	1.31 ± 0.04	18 ± 1	73 ± 15
KT	6	288 ± 10	0.13 ± 0.01	1.31 ± 0.03	17 ± 2	63 ± 9
MK + Ram	6	301 ± 20	0.14 ± 0.01	1.28 ± 0.11	17 ± 2	71 ± 17
MK	6	284 ± 32	0.13 ± 0.01	1.28 ± 0.10	18 ± 2	61 ± 10
Con	6	301 ± 24	0.13 ± 0.02	1.31 ± 0.10	16 ± 1	56 ± 11
BAY	6	308 ± 13	0.12 ± 0.01	1.32 ± 0.04	15 ± 1	68 ± 15
MK + Ram + BAY	6	287 ± 31	0.14 ± 0.02	1.27 ± 0.09	15 ± 1	60 ± 6
MK + Ram + NS	6	286 ± 11	0.13 ± 0.01	1.29 ± 0.03	17 ± 2	59 ± 9
KT + Ram + NS	6	290 ± 12	0.13 ± 0.02	1.29 ± 0.06	17 ± 1	61 ± 9

Data are mean ± SD. Con = Control; Ram = Ramelteon; KT = KT5823 (PKG inhibitor); MK = MK2206 (Akt inhibitor); BAY = BAY60-2770 (sGC activator); NS = NS1619 (K_Ca_-channel activator).

**Table 2:** Hemodynamic variables.

	Baseline	PC	Reperfusion	
			30	60
*Heart Rate (bpm)*				
Con	281 ± 34	283 ± 35	287 ± 21	265 ± 37
Ram	291 ± 62	293 ± 74	217 ± 98	206 ± 54
KT+Ram	303 ± 30	274 ± 37	211 ± 40	214 ± 54
KT	298 ± 40	275 ± 27	228 ± 75	201 ± 50
MK+Ram	288 ± 60	262 ± 74	262 ± 87	247 ± 43
MK	258 ± 32	236 ± 42	200 ± 72	232 ± 61
*Left Ventricular Developed Pressure (mmHg)*				
Con	138 ± 14	135 ± 21	29 ± 14 *	39 ± 14 *
Ram	155 ± 36	150 ± 26	23 ± 11 *	35 ± 15 *
KT+Ram	155 ± 15	148 ± 45	27 ± 19 *	33 ± 8 *
KT	145 ± 20	148 ± 34	26 ± 13 *	33 ± 15 *
MK+Ram	149 ± 37	133 ± 42	25 ± 15 *	34 ± 22 *
MK	147 ± 24	138 ± 24	31 ± 10 *	30 ± 11 *
*Coronary flow (ml/min)*				
Con	16 ± 3	17 ± 4	7 ± 2 *	6 ± 1 *
Ram	17 ± 2	16 ± 4	7 ± 2 *	6 ± 2 *
KT+Ram	16 ± 2	14 ± 2	6 ± 1 *	5 ± 1 *
KT	16 ± 1	14 ± 2	7 ± 1 *	5 ± 1 *
MK+Ram	16 ± 4	14 ± 6	7 ± 2 *	6 ± 2 *
MK	15 ± 6	13 ± 7	6 ± 2 *	5 ± 2 *

Data are mean ± SD. Con = Control; Ram = Ramelteon; KT = KT5823 (PKG inhibitor); MK = MK2206 (Akt inhibitor). * *p* < 0.0001 vs. baseline.

**Table 3:** Hemodynamic variables.

	Baseline	PC	Reperfusion	
			30	60
*Heart Rate (bpm)*				
Con	302 ± 34	299 ± 35	248 ± 65	228 ± 49
BAY	284 ± 24	262 ± 15	256 ± 67	260 ± 39
MK+Ram+BAY	276 ± 22	258 ± 32	282 ± 77	235 ± 58
MK+Ram+NS	309 ± 13	285 ± 15	260 ± 62	246 ± 47
KT+Ram+NS	288 ± 38	246 ± 29	213 ± 76	212 ± 93
*Left Ventricular Developed Pressure (mmHg)*				
Con	132 ± 20	135 ± 18	29 ± 15 *	40 ± 12 *
BAY	147 ± 18	153 ± 18	28 ± 10 *	31 ± 7 *
MK+Ram+BAY	152 ± 30	149 ± 33	22 ± 12 *	31 ± 9 *
MK+Ram+NS	147 ± 23	148 ± 25	16 ± 10 *	28 ± 16 *
KT+Ram+NS	153 ± 26	152 ± 21	19 ± 9 *	24 ± 16 *
*Coronary flow (ml/min)*				
Con	16 ± 4	16 ± 4	9 ± 1 *	8 ± 1 *
BAY	16 ± 3	16 ± 3	9 ± 1 *	8 ± 1 *
MK+Ram+BAY	15 ± 3	14 ± 3	9 ± 2 *	6 ± 1 *
MK+Ram+NS	17 ± 4	17 ± 3	7 ± 1 *	7 ± 1 *
KT+Ram+NS	17 ± 4	15 ± 2	6 ± 1 *	6 ± 1 *

Data are mean ± SD. Con = Control; BAY = BAY60-2770 (sGC activator); MK = MK2206 (Akt inhibitor); Ram = Ramelteon; NS = NS1619 (K_Ca_-channel activator); KT = KT5823 (PKG inhibitor). * *p* < 0.0001 vs. baseline.
